# Late Recurrence of Ovarian Cancer after 18 Years of Disease-Free Survival: A Case Report and Review of the Literature

**DOI:** 10.1155/2024/3938833

**Published:** 2024-01-24

**Authors:** Yoko Suzuki, Satoko Eguchi, Takahide Arimoto

**Affiliations:** ^1^Toranomon Hospital, Tokyo, Japan; ^2^University of Tokyo Hospital, Tokyo, Japan

## Abstract

We present a case of recurrent ovarian cancer at the age of 75, gravida 1 para 0, with 18 years of disease-free survival. Chemotherapy brought a 10-month partial response status; to further improve the overall survival, the patient was evaluated using the AGO score (DESKTOP III trial, 2020), which was originally intended for cases immediately after the diagnosis of recurrence; the score has indicated a significant outcome; the patient went through a hepatosplenic metastatic site resection; and complete resection was achieved. Subsequently, the PARP inhibitor was introduced, which has led to 14 months of disease-free survival. Fifteen cases of late recurrence of epithelial ovarian cancer have been reported and are summarized at the end of this paper.

## 1. Background

Late recurrence of ovarian cancer is most common in granulosa cell tumors among sex-cord stromal tumors. The recurrence often happens after 10 years. In contrast, 87-95% of epithelial ovarian cancers recur within 5 years; any recurrence beyond 10 years is rare [1]. In the present case, the patient was diagnosed with ovarian cancer recurrence after 18 years of disease-free survival (DFS). The pathological findings and the course of treatment after recurrence are presented with a discussion and review of relevant literature.

## 2. Case Presentation

The patient is 75 years old, a nulliparous woman who, 29 years ago, was diagnosed with right breast cancer and thereafter underwent mastectomy with lymphatic dissection. The pathological diagnosis was papillary ductal carcinoma, 18 mm invasion, v (+), ER (-), PgR (-), and N (0/13). The patient received postoperative adjuvant chemotherapy and radiation therapy.

During her follow-up visit which was 18 years ago, an abdominal ultrasound scan revealed a 70 mm solid-type left ovarian tumor which brought her to the obstetrics and gynecology department of our hospital.

As further examinations suggested a malignant ovarian tumor, the patient then underwent a hysterectomy, bilateral salpingo-oophorectomy, and subtotal omentectomy. The pathology was poorly differentiated endometrioid carcinoma, stage IC(b) (FIGO 1988). Five courses of TC therapy were administered after the surgery (T: Paclitaxel 175 mg/m^2^; C: Carboplatin AUC5). The patient remained recurrence-free for 10 years, and the follow-up was completed.

**Figure 1:**
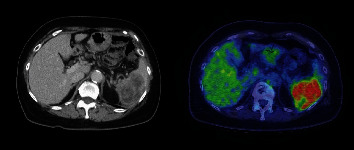
CT and PET-CT of recurrent ovarian cancer. A solid mass occupies the spleen.

At the time of 18 years after the initial treatment of ovarian cancer, following a fracture of the femoral neck, a CT scan was taken at another hospital. The scan revealed an over 70 mm mass, occupying the spleen (Figure 1). In addition, multiple maximum sizes of 10 mm tumors were seen in liver segment 6. After a liver biopsy was done, the patient was referred to the breast surgery department with a diagnosis of recurrent breast cancer.

**Figure 2:**
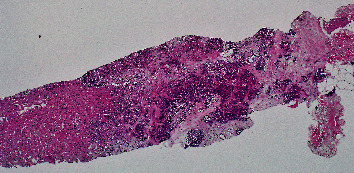
Pathology of liver metastatic site biopsy (low power field).

**Figure 3:**
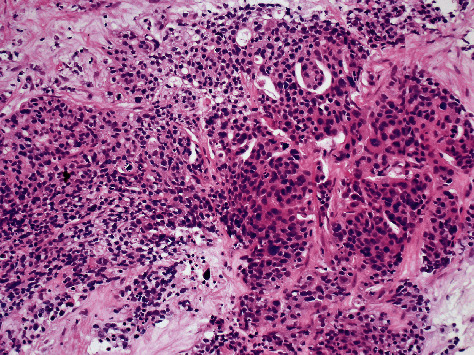
Pathology of liver metastatic site biopsy (high power field).

**Figure 4:**
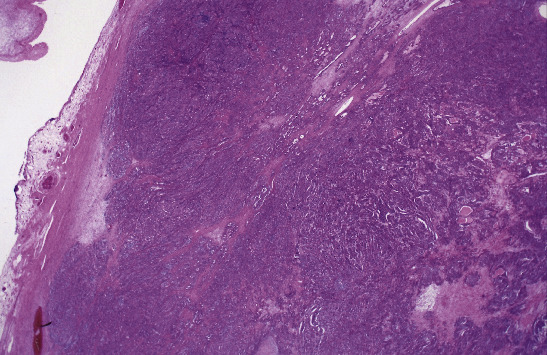
Pathology of ovarian cancer 18 years ago (low power field).

**Figure 5:**
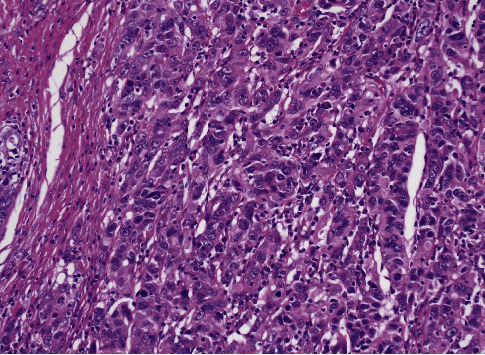
Pathology of ovarian cancer 18 years ago (high power field).

However, we were able to locate the pathology slides from 18 years ago. After histological comparison and additional immunostaining, the pathology suggested it more likely originated from the previous ovarian cancer. The specimen obtained from the liver has a histological pattern similar to that of the ovarian cancer 18 years ago. The previous ovarian cancer appeared to be a poorly differentiated endometrioid type with a solid growth pattern whereas the liver biopsy showed the proliferation of atypical cells with lumen formation adjacent to benign liver tissue. Both showed substantial growth and lumen formation (Figures 2–5).

The additional immunostaining of the liver biopsy showed the marker for breast cancer, and GCDFP15 was negative. In addition to ER (+) and PAX8 (+), immunostaining results for CK7 (+), CK20 (-), p40 (-), GCDFP15 (-), PgR (-), HER2 (-), and WT1 (+) were obtained, supporting the diagnosis of ovarian cancer recurrence. The comparison results are shown in Table 1.

When the cancer recurrence was confirmed, it could have been either breast or ovarian cancer or a new onset of peritoneal cancer. However, based on the addition of immunostaining to the biopsy of the liver metastasis, the diagnosis was made as a recurrence of ovarian cancer.

Furthermore, differentiated diagnosis in ovarian cancer histologic type, high-grade serous carcinoma (HGSC), and poorly differentiated endometrioid carcinoma could be challenging from the histological pattern alone. The immunostaining of this recurrent ovarian cancer from the liver shows WT-1 (+), which is a marker commonly seen in HGSC, suggesting that the previous ovarian cancer might have been the HGSC instead of a poorly differentiated endometrioid type.

## 3. Treatment

**Figure 6:**
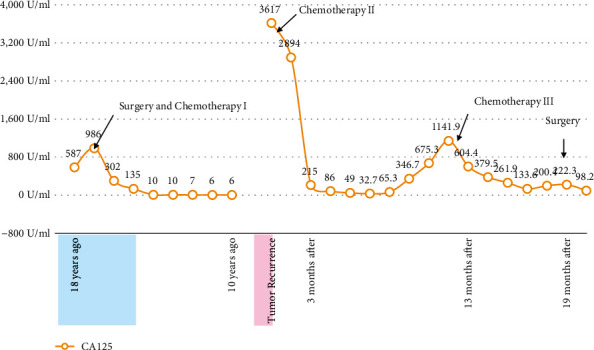
The change of CA125 during the course.

After the diagnosis of recurrence of ovarian cancer, 6 courses of TC chemotherapy were given (T: Paclitaxel 175 mg/m^2^; C: Carboplatin AUC5); thereafter, CT shows the tumor had shrunk by 64.5%, which was judged as a partial response. Tumor marker CA-125 was 3,617 U/ml at the time of recurrence and had decreased to 32.7 U/ml (Figure 6).

Maintenance therapy with bevacizumab was considered; however, during this treatment-free period, the patient suffered from a series of events: syncope attack due to bilateral middle cerebral arterial stenosis, worsening of lymphedema in the right forearm, and left total hip arthroplasty. The patient refused maintenance therapy and was under observation.

**Figure 7:**
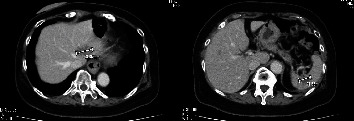
CT after 4 courses of TC therapy showed partial response. The size of the splenic tumor remained, but an increased size of the liver metastatic tumor was observed.

At 10 months of the treatment-free interval, the size of the splenic tumor remained, but an increased size of the liver metastatic tumor was observed. The patient was advised to go through a second round of TC therapy (T: Paclitaxel 135 mg/m^2^; C: Carboplatin AUC5). CT after 4 courses of TC therapy showed partial response (Figure 7). At this point, Arbeitsgemeinschaft Gynäkologische Onkologie score (AGO score), a predictor of the outcome at secondary cytoreductive surgery, was positive (ECOG performance status 0, ascites less than 500 ml, and complete gross resection recessive R0 at first surgery). After consultation with the hepatic surgery department, it was evaluated that complete resection was feasible, and surgery was planned to improve the prognosis.

**Figure 8:**
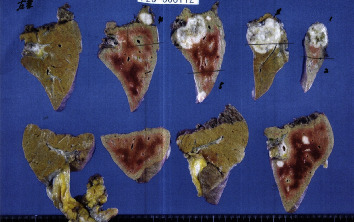
Pathology of left hepatic lobectomy and partial resections of segments 5, 6, and 8 of the right lobes.

The preoperative examination revealed severe aortic stenosis and coronary artery stenosis, so it was decided to precede the surgery with cardiovascular treatment. An immediate treatment plan of transcatheter aortic valve implantation (TAVI) and percutaneous coronary intervention (PCI) was performed. Eight weeks after the last chemotherapy, the patient was able to undergo left hepatic lobectomy and partial resections of segments 5, 6, and 8 of the right lobes (Figure 8), splenectomy, and cholecystectomy to achieve complete resection.

Due to arrhythmia and heart failure after the surgery, the patient underwent permanent pacemaker implantation. The remaining 2 courses of TC chemotherapy (T: Paclitaxel 135 mg/m^2^; C: Carboplatin AUC4) were able to begin 12 weeks postoperatively.

At this point, insurance made Olaparib available for platinum-sensitive recurrent ovarian cancer and maintenance for homologous recombination deficiency- (HRD-) positive ovarian cancer. Yet either at the time of recurrence or at the time of this new insurance coverage, the patient refused to run the BRCA analysis or the test for her HRD status. Since the consent was not obtained, these genetic tests were declined. However, considering her 18 years of platinum-free interval, she is confirmed as clinically platinum-sensitive; therefore, maintenance therapy with Olaparib (600 mg/day) was initiated. The patient has achieved 14 months of DFS with full physical status.

## 4. Discussion

Of the epithelial ovarian cancers reported so far, about 90% will recur within 4 years. Of the 214 cases reported by Sahdev et al. [1], the recurrence occurred in 67 cases, and only 4 cases recurred after 10-20 years (6%). Furthermore, there were no case reports of late recurrence with endometrioid cancer as the histologic type. According to Sahdev et al. , all were serous carcinomas, except one which was clear cell carcinoma [1]. The histology of poorly differentiated endometrioid carcinoma can be difficult to distinguish from HGSC. In this case, the immunostaining results of the metastatic lesion together with the results of the first immunostaining led to the diagnosis of recurrence of HGSC. Therefore, the histologic type of the original cancer from 18 years ago could have been HGSC.

Immunostaining ruled out the breast cancer recurrence, but differentiating from a newly onset peritoneal carcinoma remains on the table. It is widely known that for HBOC patients, post risk-reducing oophorectomy (RRSO) and BRCA1/2 gene mutation carriers are at risk of developing peritoneal carcinomatosis. The risks of developing peritoneal cancer after 5 years and 10 years were 0.3% and 0.9%, respectively [2]. The risk of developing newly onset peritoneal carcinoma is low. In addition, since there were no symptoms such as ascites nor were peritoneal seeding lesions observed during the course, it was determined that the diagnostic criteria for peritoneal cancer were not met.

Pujade-Lauraine et al. state that about 70.5% of cases are platinum-sensitive recurrence first, but almost all cases have acquired platinum resistance subsequently [3]. The problem is particularly challenging in cases of late recurrence ovarian cancer, as maximizing the control of tumors during a platinum-sensitive time is vital for prognosis.

Overall survival for recurrent ovarian cancer has long been recognized to be not significantly different between diagnoses in the early asymptomatic stage or later symptomatic stage. However, the DESKTOP II trial reported that for recurrent ovarian cancer, an AGO score could be the prediction of how feasible it is to conduct a complete resection (CGR-R0) in secondary cytoreductive surgery and to identify the cases that could benefit from performing surgery [4].

The subsequent DESKTOP III trial by Du Bois et al. compared chemotherapy with cytoreductive surgery and reported that achieving CGR-R0 will prolong both progression-free survival (PFS) and overall survival (OS) [5]. Based on this study, it is critical to diagnose the recurrence of ovarian cancer while the three AGO scores of ECOG performance status 0 (PS0), ascites less than 500 ml, and CGR-R0 at first surgery are met. A meta-analysis of the effectiveness of secondary cytoreductive surgery (SDS) in platinum-sensitive recurrent ovarian cancer was done and concluded that a maximal tumor resection in SDS will significantly contribute to the OS [6].

The patient was diagnosed with a recurrence of HGSC after 18 years of platinum-free interval, and because of the high platinum sensitivity the case has shown, we have decided to initiate the systemic treatment via platinum-contained chemotherapy and then the SDS. Although strictly speaking, the AGO score is the tool of assessment used immediately after diagnosis of recurrence from the DESKTOP III trial, the score, however, showed an effective prediction even after the administration of chemotherapy, as the patient status is improving by treatment. The patient had undergone chemotherapy first, and this brought a partial response to stable status, allowing the time for PCI and TAVI before the cytoreductive surgery, eventually achieving the best consequence CGR-R0 in SDS. In such a case, we believe the timing of assessing the AGO score is acceptable. Maintenance therapy of the PARP inhibitor was introduced, and the patient has been updating the DFS.

When the initial specimen is not suited for HRD testing, as in this case, obtaining a specimen from a recurrent site by SDS or biopsy could be a treatment strategy. Although no BRACAnalysis or HRD testing (MyChoice®) was used in this case due to the patient consent or timing of insurance coverage in Japan, testing the biomarkers for selection of the PARP inhibitor in late-recurrence platinum-sensitive cases has become increasingly important.

We have searched for reported late recurrence of epithelial ovarian cancer on PubMed and have found 15 cases in 10 reports. The characteristics of each are listed in Table 2.

## 5. Conclusion

The DESKTOP III trial reveals the importance of cytoreductive surgery, and the assessment of the potential effectiveness of SDS could be estimated by the AGO scores.

Although the patient was initially treated with chemotherapy when diagnosed with the recurrence and experienced a series of several events before the SDS, the physical status was maintained throughout the course, and the surgery has achieved complete resection, allowing the chance of introducing a new drug such as PARP inhibitors. Appropriate diagnosis and treatment strategy, including early diagnosis of recurrence, the indication of SDS, and the choice of PARP inhibitor, have become all the more important in longer survival. The patient had positive perspectives, and a shared decision was made in every treatment.

Verbal informed consent to publish the case details was obtained. Written consent forms were omitted as no data were available to identify the patient.

## Figures and Tables

**Table 1:** Immunostaining.

	CK7	CK20	ER	PgR	PAX8	Others
Liver biopsy	+	−	+	−	+	GCDDFP15(-) WT1(+)
Ovarian cancer (18 years ago)	+	−	+	−	+	N/A
Breast cancer (30 years ago)	N/A	N/A	−	−	N/A	N/A

**Table 2:** Summary of individual studies.

References	Time to recurrence (years)	Age of first treatment	Stage	Initial treatment	Histological type	Recurrence site	Treatment	DFP (months)
Zylberberg et al. [7] (3 cases)	21	33	IIIC	TAH+BSO+OM+excision of visible masses→ten cycles of monthly adjuvant chemotherapy (intraperitoneal and intravenously)	Serous	Appendix	Appendectomy, suboptimal cytoreduction, and chemotherapy	37
	21	50	IC	TAH+BSO+OM, adjuvant chemotherapy		Lower axilla, hepatic nodules, diffuse peritoneal carcinomatosis, and bone metastases	Chemotherapy	19
	26	32	IIIC	Complete cytoreduction, adjuvant chemotherapy, and radiotherapy		Para-aortic lymph node	Laparotomy excision of the mass	36

Sahdev et al. [1] (4 cases)	Ungiven, include a case of 31 years	NA	I, I, IIIC, and unknown	NA	NA	Of 4 cases, 4 pelvic mass, 2 peritoneal, 2 nodal, 2 serosal and mesenteric, 2 ascites, and visceral	Chemotherapy	NA

Testelmans et al. [8]	23	36	NA	TAH+USO and adjuvant chemotherapy	Serous papillary ovarian carcinoma	Thoracic wall	Cytoreduction of the right posterior dorsal thoracic wall, part of the diaphragm, azygos vein, thoracic duct, sympathetic chain, and mediastinal fat; mediastinal lymph node dissection; and local radiotherapy to the mediastinum and the posterior thoracic wall	12

Izuishi et al. [9]	20	32	IIC	TAH+BSO and adjuvant chemotherapy; a year later, second-look surgery, low anterior resection, and adjuvant chemotherapy	Serous papillary adenocarcinoma contained components of a poorly differentiated adenocarcinoma	Spleen	Splenectomy	60

Menczer et al. [10]	28	22	IIIC	TAH+BSO+pOM, adjuvant chemotherapy 1 year after, and “second-look” operation	Serous	Para-aortic retroperitoneal mass	Complete cytoreduction	NA

Otsuka and Shoji [11]	25	29	I	USO (right) during the first trimester of the pregnancy, spontaneous abortion after surgery, adjuvant chemotherapy, and TAH+USO (left)	High-grade endometrioid carcinoma with areas of serous histology	Para-aortic lymph node	Para-aortic lymph node resection, chemotherapy, and radiation	107

Inoue et al. [12]	29	38	III	Radical hysterectomy+OM and adjuvant chemotherapy	Serous	Lung, anterior mediastinum, and liver	VATS, chemotherapy, and subsequently bevacizumab alone	12

Omura et al. [13]	31	23	NA	BSO; 2 years later, uterine metastasis, extended hysterectomy, and adjuvant chemotherapy	Low-grade serous	Thymic epithelial tumor	Partial thymectomy	19

Chiba et al. [14]	41	38	IC	TAH+BSO+pOM and adjuvant chemotherapy	Low-grade serous	Pancreas and liver	Chemotherapy	NA

Takagi et al. [15]	30	46	NA	Radical hysterectomy and adjuvant chemotherapy	High-grade serous	Spleen, peritoneal dissemination, para-aortic and Virchow lymph nodes, intestine, and pouch of Douglas	Partial resection of the intestine; other not mentioned	NA

Current case	18	57	IC	TAH+BSO+pOM and adjuvant chemotherapy	High-grade serous	Spleen and liver	Chemotherapy, hepatic resection (right lobe; segments 5, 6, and 8+splenectomy+cholecystectomy) and adjuvant chemotherapy	56

TAH: total abdominal hysterectomy; USO: unilateral salpingo-oophorectomy; BSO: bilateral salpingo-oophorectomy; pOM: partial omentectomy; NA: none applicable.
